# Limited Diagnostic Utility of C-reactive Protein (CRP) and Erythrocyte Sedimentation Rate (ESR) for Ventriculitis in Adult and Pediatric Traumatic Brain Injury Patients With External Ventricular Drains: A Retrospective Cohort Study

**DOI:** 10.7759/cureus.106410

**Published:** 2026-04-03

**Authors:** Loui Othman, Alexandra DiGiovanni, Yahya Khan, David Millar, Claire Tse, Jacqueline DeRusso, Mark S Dias, Elias Rizk

**Affiliations:** 1 Department of Neurosurgery, Penn State College of Medicine, Hershey, USA; 2 Department of Neurosurgery, Penn State Health Milton S. Hershey Medical Center, Hershey, USA; 3 Department of Neurosurgery, The University of Texas at Austin, Austin, USA

**Keywords:** central nervous system (cns) infections, c-reactive protein (crp), erythrocyte sedimentation rate (esr), external ventricular drain (evd), traumatic brain injury (tbi), ventriculitis

## Abstract

Objective: To evaluate the diagnostic utility of erythrocyte sedimentation rate (ESR), C-reactive protein (CRP), and leukocyte count in detecting ventriculitis among adult and pediatric patients with traumatic brain injury (TBI) who underwent external ventricular drain (EVD) placement.

Methods: We performed a retrospective cohort study using the TriNetX Global Collaborative Network. Patients with TBI who underwent EVD placement were stratified by age (≥18 years vs. <18 years) and presence of ventriculitis. Propensity score matching was used to balance demographic and clinical characteristics between cohorts. Laboratory values, including ESR, CRP, leukocyte count, cerebrospinal fluid (CSF) glucose, procalcitonin, and CSF lactate, were compared using median and interquartile range (IQR) as provided by the TriNetX platform.

Results: In adults, leukocyte count, CRP, and ESR demonstrated overlapping distributions between cohorts and did not reliably distinguish ventriculitis. Although CRP reached statistical significance, values showed substantial variability and limited clinical interpretability. In the pediatric cohort, CRP, leukocyte count, and procalcitonin similarly demonstrated overlapping distributions without consistent differentiation between groups. In contrast, CSF glucose was consistently lower in patients with ventriculitis across both adult and pediatric populations. CSF lactate was higher in the adult ventriculitis cohort; however, this finding was based on a limited subset of patients and should be interpreted with caution.

Conclusion: Systemic inflammatory markers, including CRP, ESR, and leukocyte count, demonstrated limited diagnostic utility for distinguishing ventriculitis in TBI patients with EVDs. CSF-based markers, particularly CSF glucose, were more consistently associated with ventriculitis, while CSF lactate may have a potential adjunctive role. These findings support prioritizing CSF analysis and pathogen-specific testing for accurate diagnosis. Future studies should focus on prospective validation and the development of more specific biomarkers.

## Introduction

Erythrocyte sedimentation rate (ESR) and C-reactive protein (CRP) are widely used biomarkers in clinical medicine, primarily for detecting and monitoring systemic inflammation [1,2]. Their role in diagnosing infectious diseases is well known, as elevated levels often indicate bacterial or viral infections [3,4]. ESR and CRP can serve as useful co-indicators in the evaluation of ventriculitis. Elevated CRP levels have been associated with central nervous system infections and may reflect an acute-phase inflammatory response. CRP rises rapidly within 6-12 hours of infection onset and can be helpful in monitoring response to antimicrobial therapy, as declining levels often correlate with clinical improvement [4].

ESR and CRP are systemic markers that reflect an immune response to infection, inflammation, or tissue damage [5]. ESR measures the rate at which red blood cells settle in a tube over time, allowing it to serve as a general indicator of inflammation. CRP, an acute-phase reactant synthesized in the liver, rises rapidly in response to pro-inflammatory cytokines such as interleukin-6 (IL-6) and tumor necrosis factor-alpha (TNF-α) [6]. While these biomarkers are frequently elevated in bacterial infections, their ability to differentiate CNS infections from other inflammatory conditions is not without limitations. Studies have shown that serum CRP and ESR levels do not reliably distinguish between bacterial and viral meningitis, for example, often leading to indefinite diagnoses [7-9].

One major limitation of ESR and CRP in CNS infections is their lack of specificity. Elevated levels may result from a wide range of conditions, including autoimmune disorders, malignancies, and systemic inflammatory diseases [10,11]. In the context of bacterial meningitis, both ESR and CRP can be significantly increased. However, in the backdrop of other inflammatory states, their diagnostic authority diminishes [1]. Additionally, systemic inflammation does not always correlate with CSF findings. In other words, a patient with normal ESR and CRP levels can still have an active CNS infection [2]. This discrepancy challenges the utility of these markers as standalone diagnostic tools for CNS infections.

Further, CSF analysis remains the gold standard for diagnosing CNS infections [5]. Direct examination of CSF includes white blood cell count, protein, glucose levels, and pathogen-specific testing. These metrics provide more precise diagnostic information [6,12]. Unlike ESR and CRP, which reflect systemic inflammation rather than localized CNS pathology, CSF markers such as lactate and procalcitonin have been shown to be more reliable in distinguishing bacterial from viral meningitis [7]. Therefore, reliance on ESR and CRP for CNS infections may lead to delayed or inaccurate diagnoses [9].

This study aims to primarily evaluate the validity of ESR and CRP as diagnostic markers for ventriculitis. Ventriculitis is an infection-driven inflammation of the ependymal lining of the cerebral ventricular system, most commonly arising as a complication of neurosurgical procedures involving ventricular catheters or shunts but also occurring in the context of severe meningitis or brain abscesses. The diagnosis is supported by neuroimaging findings such as ependymal enhancement, intraventricular pus, debris, or septations on MRI or CT, which are considered highly suggestive in the appropriate clinical context [13]. There is a lack of consensus in the existing literature: some studies suggest these inflammatory markers may be useful, but no definitive consensus has been established regarding their reliability in diagnostics [3,14].

In addition to ESR and CRP, we will secondarily assess the diagnostic potential of other biomarkers, including leukocytes, CSF glucose, CSF lactate, and procalcitonin. Although these tests are frequently ordered in clinical practice, their validity in detecting ventriculitis remains uncertain. Using TriNetX, we aim to determine whether these biomarkers provide meaningful diagnostic value, helping to determine whether their existing widespread use in the clinical setting is warranted.

## Materials and methods

Data source and ethical considerations

This study utilized de-identified, aggregate data from the TriNetX Global Collaborative Network, a multi-institutional database comprising electronic health records from over 90 healthcare organizations. Under institutional policy, use of de-identified aggregate data was determined to be not human-subjects research and exempt from institutional review board oversight, as no Protected Health Information or individual-level identifiers were accessible. TriNetX complies with the Health Insurance Portability and Accountability Act (HIPAA) and General Data Protection Regulation (GDPR).

Study population

Patients were identified within the TriNetX platform using standardized diagnosis and procedure codes. The study included patients with traumatic brain injury (TBI) who underwent external ventricular drain (EVD) placement. Cohorts were defined as patients with TBI and EVD placement who were subsequently diagnosed with ventriculitis (cases) and patients with TBI and EVD placement without a diagnosis of ventriculitis (controls). Patients were stratified into adult (≥18 years) and pediatric (<18 years) cohorts. Inclusion criteria required documented TBI and EVD placement. Laboratory analyses were performed on available subsets of patients with recorded values within the defined analysis window.

Ventriculitis definition

Ventriculitis was defined using diagnosis codes recorded in the TriNetX database. Patients were classified as having ventriculitis if they had at least one documented diagnosis code consistent with central nervous system infection, including meningitis or encephalitis (e.g., ICD-10-CM codes G03.x, G04.x) or other specified infectious conditions (e.g., B48.x), occurring after EVD placement. Only diagnoses recorded following EVD placement were considered to preserve temporal specificity. Given the constraints of the database, microbiologic confirmation, including cerebrospinal fluid (CSF) culture results, was not consistently available and therefore not required for cohort inclusion. This definition reflects real-world diagnostic coding practices and may include clinically diagnosed or suspected cases rather than exclusively culture-confirmed infection.

Index event and laboratory window

For patients with ventriculitis, the index event was defined as the first recorded diagnosis of ventriculitis. For control patients, the index event was defined as the date of EVD placement. Laboratory values were analyzed using the most recent available measurement within a one- to seven-day period following the index event, consistent with data availability within the TriNetX platform. If multiple laboratory values were present within this window, the value closest to the index event was selected for analysis. This approach reflects real-world clinical timing of diagnosis and was necessary given the structure of the TriNetX database.

Study period

The study included patient encounters recorded within the TriNetX network over an approximate 20-year period, with all queries executed within the platform at the time of analysis.

Outcome measures

The primary outcome was the presence of ventriculitis. Laboratory markers evaluated included leukocyte count, CRP, ESR, CSF glucose, and serum procalcitonin. Laboratory measurements were reported in standard clinical units: CRP (mg/L), ESR (mm/h), leukocyte count (×10^3^/µL), procalcitonin (ng/mL), and CSF glucose (mg/dL). Cytokine-based markers (e.g., IL-6, interleukin-1β (IL-1β), TNF-α, and interleukin-10 (IL-10)) were not included in the final analysis due to insufficient data availability within the database.

Data analysis

Propensity score matching was performed to reduce confounding between cohorts, matching patients based on age, sex, race/ethnicity, and Glasgow Coma Scale (GCS) score. Although GCS was included in the matching process to account for injury severity, detailed reporting was limited, particularly in the pediatric cohort, due to sparse data and small sample sizes, which reduced interpretability despite adequate balance following matching.

All analyses were conducted using the TriNetX analytics platform, which provides de-identified aggregate counts and summary statistics without access to individual-level data. Continuous variables were summarized using mean ± standard deviation and median with interquartile range (IQR), as provided directly by the TriNetX platform. Given the observed variability and skewness in laboratory values, the median and IQR were emphasized for descriptive interpretation. Between-group comparisons for continuous variables were performed using t-tests as provided within the TriNetX platform. A two-sided p-value <0.05 was considered statistically significant.

## Results

Following propensity score matching, the adult (≥18 years) cohorts each consisted of 1,662 patients with TBI who underwent EVD placement. Cohort 1 included patients diagnosed with ventriculitis, while Cohort 2 included patients without ventriculitis.

Baseline demographic characteristics were well balanced between groups after matching. The mean age at index was 46.9 ± 19.0 years in Cohort 1 and 46.2 ± 19.3 years in Cohort 2, with similar distributions across both cohorts. Sex, race, and ethnicity distributions were comparable, and no clinically meaningful differences were observed. GCS categories were also balanced, although overall representation across categories was limited. These findings are summarized in Table 1.

**Table 1:** Demographic and clinical characteristics of adults with traumatic brain injury (TBI) who underwent external ventricular drain (EVD) placement. Propensity score matching was performed using age, sex, race/ethnicity, and GCS. Values are presented as mean ± standard deviation or n (%). Standardized mean differences (SMDs) are reported to assess covariate balance, with values <0.1 indicating adequate balance. Percentages may not sum to 100% due to rounding. GCS data were limited and may not reflect the full cohort.

Characteristic	Ventriculitis (n = 1,662)	No Ventriculitis (n = 1,662)	Standardized Mean Difference
Age, years (mean ± SD)	46.9 ± 19.0	46.2 ± 19.3	0.038
Sex, n (%)			
Male	1,051 (63.2)	1,062 (63.9)	0.014
Female	610 (36.7)	600 (36.1)	0.013
Race, n (%)			
White individuals	938 (56.4)	940 (56.6)	0.002
Black or African American individuals	291 (17.5)	299 (18.0)	0.013
Asian individuals	100 (6.0)	105 (6.3)	0.013
Other	89 (5.4)	85 (5.1)	0.011
Unknown	217 (13.1)	211 (12.7)	0.011
Ethnicity, n (%)			
Hispanic or Latino individuals	177 (10.6)	169 (10.2)	0.016
Not Hispanic or Latino individuals	1,188 (71.5)	1,195 (71.9)	0.009
Unknown	297 (17.9)	298 (17.9)	0.002
Glasgow Coma Scale (GCS), n (%)			
13-15	48 (2.9)	39 (2.3)	0.034
9-12	46 (2.8)	34 (2.0)	0.047
3-8	129 (7.8)	110 (6.6)	0.044

Similarly, in the pediatric population (<18 years), propensity score matching yielded 181 patients in each cohort. Baseline characteristics, including age, sex, race, and ethnicity, were well balanced between groups with no clinically meaningful differences observed. GCS data were limited across categories and were not considered meaningful for interpretation despite inclusion in the matching process. These findings are summarized in Table 2.

**Table 2:** Demographic and clinical characteristics of pediatric patients (<18 years) with traumatic brain injury (TBI) who underwent external ventricular drain (EVD) placement, after propensity score matching. Propensity score matching was performed using age, sex, race/ethnicity, and Glasgow Coma Scale (GCS). Values are presented as mean ± standard deviation or n (%). Standardized mean differences (SMD) are reported to assess covariate balance, with values <0.1 indicating adequate balance. Percentages may not sum to 100% due to rounding. GCS was included in the matching process to account for injury severity; however, detailed categorical reporting was limited due to sparse data in the pediatric cohort.

Characteristic	Ventriculitis (n = 181)	No Ventriculitis (n = 181)	Standardized Mean Difference
Age, years (mean ± SD)	5.6 ± 4.9	5.5 ± 4.8	0.011
Sex, n (%)			
Male	106 (58.6)	105 (58.0)	0.011
Female	75 (41.4)	76 (42.0)	0.011
Race, n (%)			
White individuals	97 (53.6)	105 (58.0)	0.089
Black or African American individuals	37 (20.4)	30 (16.6)	0.100
Asian individuals	10 (5.5)	10 (5.5)	<0.001
Unknown	28 (15.5)	28 (15.5)	<0.001
Ethnicity, n (%)			
Hispanic or Latino individuals	34 (18.8)	33 (18.2)	0.014
Not Hispanic or Latino individuals	124 (68.5)	125 (69.1)	0.012
Unknown	23 (12.7)	23 (12.7)	<0.001

Inflammatory laboratory markers

Across both age groups, most cytokine-based markers, including IL-6, IL-1β, TNF-α, TGF-β, and IL-10, had no recorded values within the defined time window and were excluded from further analysis. The primary analytes with sufficient data included leukocyte count, CRP, and ESR.

Leukocyte count was available in 1,145 patients with ventriculitis and 1,220 patients without. Median leukocyte values were similar between groups (9.39 vs. 10.50) with overlapping distributions, and no statistically significant difference was observed (p = 0.123).

CRP values were available in 274 ventriculitis patients and 193 non-ventriculitis patients. Median CRP levels were lower in the ventriculitis cohort (29 vs. 79.2); however, values demonstrated substantial variability with broad and overlapping distributions. This difference reached statistical significance (p < 0.001) but remained of limited clinical interpretability.

ESR was recorded in 125 ventriculitis patients and 27 non-ventriculitis patients. Median ESR values were comparable between groups (47 vs. 44) with overlapping IQRs and no statistically significant difference (p = 0.558). These findings are summarized in Table 3.

**Table 3:** Inflammatory markers in adults (≥18 years). Values are presented as median and interquartile range (IQR). “Patients (C1/C2)” reflects the number of patients with available laboratory data in each cohort. Laboratory availability varied and may reflect clinically indicated testing, introducing potential selection bias. * indicates statistical significance (p < 0.05).

Biomarker	Patients (C1/C2)	Median (C1)	IQR (C1)	Median (C2)	IQR (C2)	p-value
Leukocyte count (×10^3^/µL)	1,145/1,220	9.39	5.62	10.50	5.20	0.123
C-reactive protein (mg/L)	274/193	29.0	88.0	79.2	115.3	<0.001*
Erythrocyte sedimentation rate (mm/h)	125/27	47	51	44	64	0.558

Additional biomarkers

In the adult cohort, CSF glucose was available in 659 patients with ventriculitis and 459 without, with lower median values observed in the ventriculitis group (68 vs. 81, p < 0.001). Procalcitonin values were available in a subset of patients and demonstrated substantial variability with overlapping distributions between cohorts, without a statistically significant difference (p = 0.116).

CSF lactate was available in 53 patients with ventriculitis and 44 without, with higher median values observed in the ventriculitis cohort (4.1 vs. 2.6, p = 0.028). However, sample sizes were limited, and values demonstrated variability across groups. These findings are summarized in Table 4.

**Table 4:** Cerebrospinal fluid (CSF) biomarkers in adult patients (≥18 years). Secondary biomarkers demonstrated substantial variability across cohorts. CSF glucose was lower in the ventriculitis cohort and showed the most consistent association. In contrast, CSF lactate was higher in the ventriculitis cohort, while procalcitonin demonstrated overlapping distributions without a statistically significant difference between groups. Values are presented as median and interquartile range (IQR). “Patients (C1/C2)” reflects the number of patients with available data in each cohort. Laboratory availability varied across cohorts and reflects clinically obtained measurements, which may introduce selection bias. Units are reported as provided within the TriNetX platform. * indicates statistical significance (p < 0.05).

Biomarker	Patients (C1/C2)	Median (C1)	IQR (C1)	Median (C2)	IQR (C2)	p-value
CSF Glucose (mg/dL)	659/459	68	31	81	24	<0.001*
Procalcitonin (ng/mL)	177/177	0.20	0.46	0.22	0.63	0.116
CSF Lactate (mmol/L)	53/ 44	4.10	3.30	2.60	1.90	0.028*

Pediatric population (<18 years)

In the pediatric population (<18 years), biomarker analysis included CRP, procalcitonin, leukocyte count, and CSF glucose based on data availability.

CRP was available in 58 patients with ventriculitis and 22 without, with median values of 26.8 and 28.0, respectively. Although this difference reached statistical significance (p = 0.018), the values demonstrated substantial variability with overlapping IQRs.

Procalcitonin was available in 21 ventriculitis patients and 15 non-ventriculitis patients, with median values of 0.17 and 0.24, respectively. No statistically significant difference was observed (p = 0.146), and values again demonstrated considerable variability across cohorts.

Leukocyte count was available in 119 ventriculitis patients and 114 non-ventriculitis patients, with median values of 10.3 and 9.94, respectively. No statistically significant difference was observed (p = 0.231), with overlapping distributions between groups.

CSF glucose was available in 82 ventriculitis patients and 64 non-ventriculitis patients, with lower median values observed in the ventriculitis cohort (59 vs. 70, p < 0.001).

ESR and CSF lactate data were not available in sufficient quantity in the updated analysis and were therefore not included. These findings are summarized in Table 5.

**Table 5:** Inflammatory and CSF biomarkers in pediatric patients (<18 years). Values are presented as median and interquartile range (IQR). “Patients (C1/C2)” reflects the number of patients with available data in each cohort. Laboratory availability varied across biomarkers and reflects clinically obtained measurements, which may introduce selection bias. ESR data were not available in sufficient quantity in the updated analysis and were not included. * indicates statistical significance (p < 0.05). ESR: erythrocyte sedimentation rate; CSF: cerebrospinal fluid

Biomarker	Patients (C1/C2)	Median (C1)	IQR (C1)	Median (C2)	IQR (C2)	p-value
C-reactive protein (mg/L)	58/22	26.8	41.0	28.0	141.1	0.018*
Procalcitonin (ng/mL)	21/15	0.17	0.36	0.24	2.81	0.146
Leukocyte count (×10^3^/µL)	119/114	10.3	5.83	9.94	4.28	0.231
CSF Glucose (mg/dL)	82/64	59	21	70	22	<0.001*

Summary of findings

Across both adult and pediatric populations, systemic inflammatory markers, including CRP, ESR, and leukocyte count, demonstrated substantial variability and limited ability to distinguish between ventriculitis and non-ventriculitis cohorts. Although CRP reached statistical significance in select comparisons, its broad dispersion and overlapping distributions limit its clinical utility.

Procalcitonin similarly demonstrated considerable variability and did not reliably differentiate between cohorts. In contrast, CSF glucose was consistently lower in patients with ventriculitis across both populations and represented the most reliable biomarker associated with the diagnosis. CSF lactate was elevated in the ventriculitis cohort in the adult population; however, this finding was based on a limited subset of patients and should be interpreted with caution.

Taken together, these findings suggest that commonly used systemic inflammatory markers have limited diagnostic utility in this setting, while CSF-based markers, particularly CSF glucose, and potentially CSF lactate, may provide greater clinical value for distinguishing ventriculitis.

## Discussion

This retrospective study investigated the utility of using inflammatory biomarkers for the assessment and treatment of patients who present with TBIs requiring extra-ventricular drainage that secondarily causes an infection. An EVD for the treatment of a TBI increases the patient’s risk of developing ventriculitis, which necessitates the availability of quick, reliable diagnostic indicators to assess the development of infection [10]. A consequent infection is thought to occur, on average, in greater than 10% of cases [15]. Ventricular lavage and irrigation with antibiotics have been shown to benefit outcomes in individuals with ventriculitis, making proper diagnosis and timely treatment essential for ensuring effective delivery and quality of care [16]. Importantly, CSF infections must be appropriately screened to prevent the sequelae that can occur with treatment delay, which contributes to worsened patient outcomes.

The findings of this study suggest that using many commonly assessed inflammatory biomarkers does not adequately distinguish between those with or without ventriculitis following EVD insertion, thereby indicating these are not adequate screening tools to assess infection in TBI patients after initial drainage interventions that have an associated infection risk.

A 2021 study reported an in-hospital mortality rate of approximately 30% among patients with ventriculitis, and in patients who survived, neurologic decline and functional impairment were common, highlighting the severity of this condition [17,18]. Importantly, the clinical context of EVD placement must be considered when evaluating systemic inflammatory markers. Patients requiring EVDs are often critically ill, and systemic inflammation may already be elevated due to trauma or other intensive care-related processes [10]. This background elevation reduces the diagnostic specificity of markers like CRP and ESR, further limiting their utility in detecting localized infections such as ventriculitis. Given their affordability and accessibility, these markers are often considered mainstays in a diagnostic workup, particularly where infection or inflammation is suspected. Over the past five years, however, numerous meta-analyses have sought to assess the sensitivity and specificity of these markers for various infectious conditions and have nearly ubiquitously indicated limited diagnostic accuracy [8].

In the adult cohorts, ESR did not differ significantly between patients with and without ventriculitis. However, CRP was higher in the non-ventriculitis group, which may reflect the fact that CRP is a marker of systemic inflammation. Ventriculitis, being a relatively localized CNS infection, may not provoke as strong a systemic inflammatory response, whereas patients without ventriculitis may have other systemic inflammatory or infectious processes contributing to elevated CRP levels. When pediatric cohorts were assessed, the CRP and ESR differences were statistically insignificant. These findings further call into question the reliability of ESR and CRP as prognostic indicators for ventriculitis following EVD placement in patients with TBI.

Additionally, leukocyte counts in blood samples were not found to be a useful indicator of ventriculitis, as no statistically significant differences were observed between cohorts in either adult or pediatric populations. Taken together, these results highlight the need for improved and more reliable diagnostic approaches.

Assessment of other inflammatory markers, such as cytokines commonly involved in inflammatory processes, including IL-6, IL-6 in CSF, IL-1β, TGF-β, TNF-α, and IL-10, was not possible, as no data have been recorded for the patients in the TriNetX database. The absence of data for these markers across all assessed patients in both adult and pediatric cohorts indicates a lack of real-world testing of these inflammatory indicators in the context of ventriculitis evaluation. Similar to CRP, even if these markers had been evaluated, their utility in assessing ventriculitis development would likely be limited, given that they can reflect other inflammatory or infectious processes and lack sufficient diagnostic specificity [13,19].

Additional biomarkers evaluated for their diagnostic value in ventriculitis included serum procalcitonin and CSF lactate. Procalcitonin did not demonstrate statistically significant differences between cohorts in either adult or pediatric populations and showed substantial variability with overlapping distributions. These findings contrast with a prior study that reported an association between ventriculitis and elevated procalcitonin levels following EVD placement; however, that study was limited by a small sample size of 34 patients [20]. In contrast, other studies have similarly reported that procalcitonin is not a reliable diagnostic marker for ventriculitis, which is consistent with the findings of the present analysis [21].

CSF lactate was elevated in the ventriculitis cohort in the adult population, suggesting a potential association with infection-related metabolic changes. However, this finding was based on a limited subset of patients and should be interpreted with caution. Further investigation is warranted to determine the potential role of CSF lactate as an adjunctive biomarker in the diagnosis of ventriculitis.

The results of these analyses are consistent with much of the existing literature, which suggests that direct CSF examination and pathogen-specific testing remain the most effective methods for the accurate and timely diagnosis of ventriculitis [5,6]. However, further exploration of alternative diagnostic approaches is warranted, as CSF extraction and analysis are not without risk, particularly in vulnerable populations such as patients with TBI.

Limitations

This study has several important limitations. First, its retrospective design introduces the potential for residual confounding despite the use of propensity score matching. Second, the use of the TriNetX database limits analysis to de-identified, aggregate-level data without access to individual patient-level values, precluding detailed data validation, unit harmonization across contributing sites, and granular assessment of outliers or data distributions.

Third, laboratory availability varied across biomarkers and likely reflects clinically indicated testing rather than systematic sampling, introducing potential selection bias and limiting generalizability. This was particularly relevant in the pediatric cohort, where smaller sample sizes reduced statistical power for certain comparisons. Additionally, variability in clinical practice patterns, timing of laboratory acquisition relative to EVD placement, and diagnostic thresholds across participating institutions may have introduced heterogeneity into the dataset.

Finally, the use of diagnosis codes to define ventriculitis may include clinically suspected cases rather than exclusively culture-confirmed infections, which may affect classification accuracy. These factors should be considered when interpreting the diagnostic performance of systemic inflammatory markers in this analysis.

## Conclusions

In this retrospective cohort analysis, systemic inflammatory markers, including CRP, ESR, and leukocyte count, demonstrated limited ability to distinguish ventriculitis in patients with TBI requiring EVD. These findings should be interpreted within the constraints of aggregate-level data and variable biomarker availability, which may influence precision and generalizability. Clinically, these results support prioritizing CSF analysis and pathogen-specific testing for accurate diagnosis. Future research should focus on prospective study designs and the identification of more specific biomarkers to improve early detection in both adult and pediatric populations.

## References

[1] Bray C, Bell LN, Liang H, Haykal R, Kaiksow F, Mazza JJ, Yale SH (2016). Erythrocyte sedimentation rate and C-reactive protein measurements and their relevance in clinical medicine. WMJ.

[2] Sindic CJ, Collet-Cassart D, Depré A, Laterre EC, Masson PL (1984). C-reactive protein in serum and cerebrospinal fluid in various neurological disorders. Apparent local consumption during bacterial meningitis. J Neurol Sci.

[3] Lenski M, Biczok A, Neufischer K, Tonn JC, Briegel J, Thon N (2019). Significance of cerebrospinal fluid inflammatory markers for diagnosing external ventricular drain-associated ventriculitis in patients with severe traumatic brain injury. Neurosurg Focus.

[4] Póvoa P, Almeida E, Moreira P, Fernandes A, Mealha R, Aragão A, Sabino H (1998). C-reactive protein as an indicator of sepsis. Intensive Care Med.

[5] Aronin SI, Peduzzi P, Quagliarello VJ (1998). Community-acquired bacterial meningitis: risk stratification for adverse clinical outcome and effect of antibiotic timing. Ann Intern Med.

[6] van de Beek D, Cabellos C, Dzupova O (2016). ESCMID guideline: diagnosis and treatment of acute bacterial meningitis. Clin Microbiol Infect.

[7] Brouwer MC, Tunkel AR, van de Beek D (2010). Epidemiology, diagnosis, and antimicrobial treatment of acute bacterial meningitis. Clin Microbiol Rev.

[8] Spellberg B, Nielsen TB, Phillips MC (2025). Revisiting diagnostics: erythrocyte sedimentation rate and C-reactive protein: it is time to stop the zombie tests. Clin Microbiol Infect.

[9] Diculencu D, Miftode E, Turcu T, Buiuc D (1995). The value of C-reactive protein for the differentiation of bacterial meningitis from viral meningitis [Article in Romanian]. Rev Med Chir Soc Med Nat Iasi.

[10] Dubos F, Korczowski B, Aygun DA (2008). Serum procalcitonin level and other biological markers to distinguish between bacterial and aseptic meningitis in children: a European multicenter case cohort study. Arch Pediatr Adolesc Med.

[11] Brigden M (1998). The erythrocyte sedimentation rate. Still a helpful test when used judiciously. Postgrad Med.

[12] Gomes HR (2022). Cerebrospinal fluid analysis: current diagnostic methods in central nervous system infectious diseases. Arq Neuropsiquiatr.

[13] Allos H, Hasbun R (2024). Current understanding of infection of the ventricles and its complications. Expert Rev Anti Infect Ther.

[14] Assasi N, Blackhouse G, Campbell K (2015). Comparative Value of Erythrocyte Sedimentation Rate (ESR) and C-Reactive Protein (CRP) Testing in Combination Versus Individually for the Diagnosis of Undifferentiated Patients With Suspected Inflammatory Disease or Serious Infection: A Systematic Review and Economic Analysis. https://www.ncbi.nlm.nih.gov/books/NBK333366/.

[15] Santos Araujo AB, Gusmão S, Magaldi M, Vanderlei AS, Ribeiro Cambraia MB (2013). Risk factors for infection in external ventricular drains. Arq Bras Neurocir.

[16] Walek KW, Leary OP, Sastry R, Asaad WF, Walsh JM, Horoho J, Mermel LA (2022). Risk factors and outcomes associated with external ventricular drain infections. Infect Control Hosp Epidemiol.

[17] Luque-Paz D, Revest M, Eugène F, Boukthir S, Dejoies L, Tattevin P, Le Reste PJ (2021). Ventriculitis: a severe complication of central nervous system infections. Open Forum Infect Dis.

[18] Karvouniaris M, Brotis A, Tsiakos K, Palli E, Koulenti D (2022). Current perspectives on the diagnosis and management of healthcare-associated ventriculitis and meningitis. Infect Drug Resist.

[19] Kany S, Vollrath JT, Relja B (2019). Cytokines in inflammatory disease. Int J Mol Sci.

[20] Berger C, Schwarz S, Schaebitz WR, Aschoff A, Schwab S (2002). Serum procalcitonin in cerebral ventriculitis. Crit Care Med.

[21] Martínez R, Gaul C, Buchfelder M, Erbguth F, Tschaikowsky K (2002). Serum procalcitonin monitoring for differential diagnosis of ventriculitis in adult intensive care patients. Intensive Care Med.

